# Supplementary material for: Chromothripsis during telomere crisis is independent of NHEJ, and consistent with a replicative origin
Source: Genome Res. 2019 May;29(5):737–49. doi: 10.1101/gr.240705.118 (PMC6499312; doi:10.1101/gr.240705.118)
Supplement: Supplemental Material [file supp_gr.240705.118_Supplemental_file_1.zip › contigs/annotated_contigs/DB51/contig.2.DB51_length_685_mean_cov_5.64817518248.docx]

**DB51_length_685_mean_cov_5.64817518248**

```
ATTTCACTATTTCATTATGCCTATTCAAATGATCTCAGTCCTTGCCAGCTCCTTACAGCCTCCATTCTTTGCTGGAAATACCCTTATCC
 >chr6:10518657-10518987 - E=9e-187 p=0e+00
TTTCACCTCCTGGCCACCCAACCTGTTCTTCTCCATCACAAACAATAGAGCATTGAAATTCTGTGCCTGCCACCCAGAAAACTGGCAAC

CCACAAAGAACAAGAAAAATGGCTAACATACCTTTAAAATATATTCATCTTTAACAAATTATAAATAACAAGAGTAGCGCAGTTCTTTT

TCTGTTTAATTCAAGTAATTTTTTAAAAGAAATACATTAGGCCAGGTGCGGTGGCTCACGCCT|ATAGCCAGGTGCGG|TGGCTTGTTT
                                                                              >chr4:112
CTATTTATATTACTTTGCTTACGAGTAAATGTATAAAGAGTTTGAAGCTCAGAGACTAAATTTGCAGATATCATTTAGACCTTAATTTG
374925-112375266 + E=2e-187
ATATTTATATCTTGGATCATGTAGAGAAGTCTGGTTTTGACAGAAAATTACATGCTTGCTATCCATTTATGATACTGCATTCATTATAA

TGTAATCTTTGCTATGTTGTATGCATTACATTGTTGTTATATTTTATGTATGATATAATCAATAAAGAAATAATAAAAATAATTCTGAA

TGACTGTCTTGAACTGTTTGTACTTCAGCTGGACTATTTAGCAGTGAGTTAAAGCCTCTTTATT
```
